# Prevalence of Antibiotic-Resistant Pathogenic Bacteria and Level of Antibiotic Residues in Hospital Effluents in Selangor, Malaysia: Protocol for a Cross-sectional Study

**DOI:** 10.2196/39022

**Published:** 2023-05-29

**Authors:** Sophia Karen Bakon, Zuraifah Asrah Mohamad, Mohd Azerulazree Jamilan, Hazimah Hashim, Mohamed Yazid Kuman, Rafiza Shaharudin, Norazah Ahmad, Nor Asiah Muhamad

**Affiliations:** 1 Environmental Health Research Centre Institute for Medical Research National Institutes of Health, Ministry of Health Malaysia Shah Alam Malaysia; 2 Nutritional, Metabolic and Cardiovascular Research Centre Institute for Medical Research National Institutes of Health, Ministry of Health Malaysia Shah Alam Malaysia; 3 Pharmacy Practice and Development Division Ministry of Health Malaysia Petaling Jaya Malaysia; 4 Engineering Service Division Ministry of Health Malaysia Putrajaya Malaysia; 5 Infectious Disease Research Centre Institute for Medical Research National Institutes of Health, Ministry of Health Malaysia Shah Alam Malaysia; 6 Evidence Based Unit National Institutes of Health, Ministry of Health Malaysia Shah Alam Malaysia

**Keywords:** ESKAPE, antimicrobial resistance, hospital effluent, antibiotics, health care, antibiotic resistance, antimicrobial, hospital setting, antibiotic residues, wastewater

## Abstract

**Background:**

Antimicrobial resistance (AMR) has emerged as a major global public health challenge due to the overuse and misuse of antibiotics for humans and animals. Hospitals are among the major users of antibiotics, thereby having a large contribution to AMR.

**Objective:**

The aim of this study is to determine the prevalence of antibiotic-resistant pathogenic bacteria and the level of antibiotic residues in the hospital effluents in Selangor, Malaysia.

**Methods:**

A cross-sectional study will be performed in the state of Selangor, Malaysia. Tertiary hospitals will be identified based on the inclusion and exclusion criteria. The methods are divided into three phases: sample collection, microbiological analysis, and chemical analysis. Microbiological analyses will include the isolation of bacteria from hospital effluents by culturing on selective media. Antibiotic sensitivity testing will be performed on the isolated bacteria against ceftriaxone, ciprofloxacin, meropenem, vancomycin, colistin, and piperacillin/tazobactam. The identification of bacteria will be confirmed using 16S RNA polymerase chain reaction (PCR) and multiplex PCR will be performed to detect resistance genes (*ermB*, *mecA*, *bla_NDM-L_*, *bla_CTX-M_*, *bla_OXA-48_*, *bla_SHV_*, *VanA*, *VanB*, *VanC1*, *mcr-1*, *mcr-2*, *mcr-3*, *Intl1*, *Intl2,* and *qnrA*). Finally, the level of antibiotic residues will be measured using ultrahigh-performance liquid chromatography.

**Results:**

The expected outcomes will be the prevalence of antibiotic-resistant *Enterococcus faecium*, *Staphylococcus aureus*, *Klebsiella pneumoniae*, *Acinetobacter baumannii*, *Pseudomonas aeruginosa*, and *Enterobacter* (ESKAPE) bacterial species from the hospital effluents, the occurrence of antibiotic resistance genes (ARGs) from the isolated ESKAPE bacteria, and the level of antibiotic residues that may be detected from the effluent. Sampling has been conducted in three hospitals. Data analysis from one hospital showed that as of July 2022, 80% (8/10) of *E. faecium* isolates were resistant to vancomycin and 10% (1/10) were resistant to ciprofloxacin. Further analysis will be conducted to determine if the isolates harbor any ARGs and effluent samples are being analyzed to detect antibiotic residues. Sampling activities will be resumed after being suspended due to the COVID-19 pandemic and are scheduled to end by December 2022.

**Conclusions:**

This study will provide the first baseline information to elucidate the current status of AMR of highly pathogenic bacteria present in hospital effluents in Malaysia.

**International Registered Report Identifier (IRRID):**

DERR1-10.2196/39022

## Introduction

### Background

Antibiotics have been the most crucial medications for the treatment of infections since the 1940s. However, some bacteria have developed resistance to practically all of the widely accessible antibiotics, which makes them capable of causing serious illness, resulting in a significant public health issue. Due to the selective pressure caused by the overuse of antibiotics, antibiotic-resistant bacteria (ARB) have developed and spread among human and animal populations worldwide [1].

The overuse and abuse of antibiotics have contributed to the global epidemic of antimicrobial resistance (AMR). Environmental contamination with antibiotic residues and resistant microorganisms and genes due to human activity has been demonstrated in pharmaceutical plants and may be a leading driver of the spread of ARB. Insufficient wastewater management by bulk drug manufacturing facilities has led to unprecedented levels of contamination of water resources with antimicrobial pharmaceuticals, which seems to be associated with the selection and dissemination of carbapenemase-producing pathogens [2]. In India, the concentration of the most abundant drug, ciprofloxacin (up to 31,000 μg/L), was reported to exceed the toxicity level by over 1000-fold [3].

Several hospital-acquired outbreaks caused by gram-negative bacteria such as *Acinetobacter baumannii* and *Pseudomonas aeruginosa* have been associated with contaminated water distribution systems [4-6]. The presence of *Escherichia coli* has been used as an indicator of fecal contamination in drinking water. Multidrug-resistant *E. coli* have been isolated from food sources [7] and urinary samples [8], as well as from hospital and municipal effluents [9]. However, the relationship between environmentally driven and clinically acquired resistant bacteria remains unclear; therefore, it is important to determine whether this effect is either strictly environmental or clinical.

The hospital setting has been the focus of AMR as one of the primary sources owing to the high usage of a wide range of antibiotics and the spread of antibiotic resistance genes (ARGs) that may be transferred by the ARB released into the environment from hospital effluents [10]. Some antibiotic-resistant pathogens such as methicillin-resistant *Staphylococcus aureus* (MRSA), carbapenem-resistant Enterobacteriaceae (CRE), and vancomycin-resistant enterococci (VRE) may pose significant risks to human health [11-13]. Ignorance that these possible effluent sources such as hospitals, including ARB or ARGs from clinical sources, can be dispersed and even thrive in the environment is likely to accelerate the development of multiple antibiotic–resistant bacteria.

Although antibiotics remain effective at treating many bacterial infections, some strains are extremely difficult to treat, and therapeutic options are becoming increasingly scarce. The term “ESKAPE” encompasses six such pathogens with growing multidrug resistance and virulence: *Enterococcus faecium*, *Staphylococcus aureus*, *Klebsiella pneumoniae*, *A. baumannii*, *P. aeruginosa*, and *Enterobacter* species [14]. ESKAPE pathogens are responsible for most nosocomial infections and are capable of resisting the biocidal action of antimicrobial agents. This phenomenon is caused by bacterial evolution; thus, AMR has increasingly become a problem with rapid rates of development and spread, and we are lacking new drugs to challenge these new superbugs.

The World Health Organization (WHO) has called for coordinated actions to minimize the emergence and spread of AMR by providing technical assistance for countries to develop national health action plans and urging more research and development on AMR [15]. In Malaysia, the collaborative effort between the Ministry of Health and the Ministry of Agriculture and Agro-based Industry resulted in the Malaysian Action Plan on Antimicrobial Resistance (MyAP-AMR) 2017-2021. The MyAP-AMR details plans to minimize AMR in both health care practices and agricultural sectors, including increasing awareness of antimicrobials, appropriate usage of antibiotics in clinical settings, and pollution influenced by the direct use of antibiotics in poultry farms [16].

The Bacteriology Unit, Infectious Diseases Research Centre in the Institute for Medical Research has performed profiling of antibiotic resistance from a poultry farm and clinical samples in Malaysia. Some published studies also reported the presence of pharmaceutical residues from surface water in Malaysia. Al-Odaini et al [17] documented the presence of human pharmaceutical products—including antidiabetic, antihypertensive, and hypolipidemic agents; β2-adrenergic receptor agonists; antihistamines; analgesics; and sex hormones—in river water and sewage effluents, although no antibiotics were measured. Praveena et al [18] reported the presence of pharmaceutical residues, including antibiotics, in three rivers in Selangor, Malaysia. The researchers detected ciprofloxacin in all samples, which was the most common pollutant in the rivers. Recently, Bong et al [19] found that multiple antibiotic-resistant phenotypes of *E. coli*, harboring the *tet* and *sul* resistance genes, were more prevalent in wastewater effluents than in the river waters. A concerted effort has been carried out to address the issue of AMR in clinical settings and the misuse of antibiotics in the current practice of poultry farming and pharmaceutical manufacturing industries. However, Malaysia lacks research on environmental aspects that may contribute to AMR, particularly the possibility of ARB and ARGs that may be released from hospitals into the environment through various modes. Moreover, no study has investigated whether antibiotic residues present in the hospital effluent contribute to the AMR problem in Malaysia. Since hospitals are the major users of antibiotics, it is important to determine the status of the effluent water from our hospitals that is released into the environment. However, to date, there has been no monitoring of the antibiotic residues under wastewater management in Malaysia. Therefore, this proposed study represents a first step to fill this gap to better address the AMR issues in Malaysia.

### Dissemination of Antibiotic Resistance

**Figure 1:**
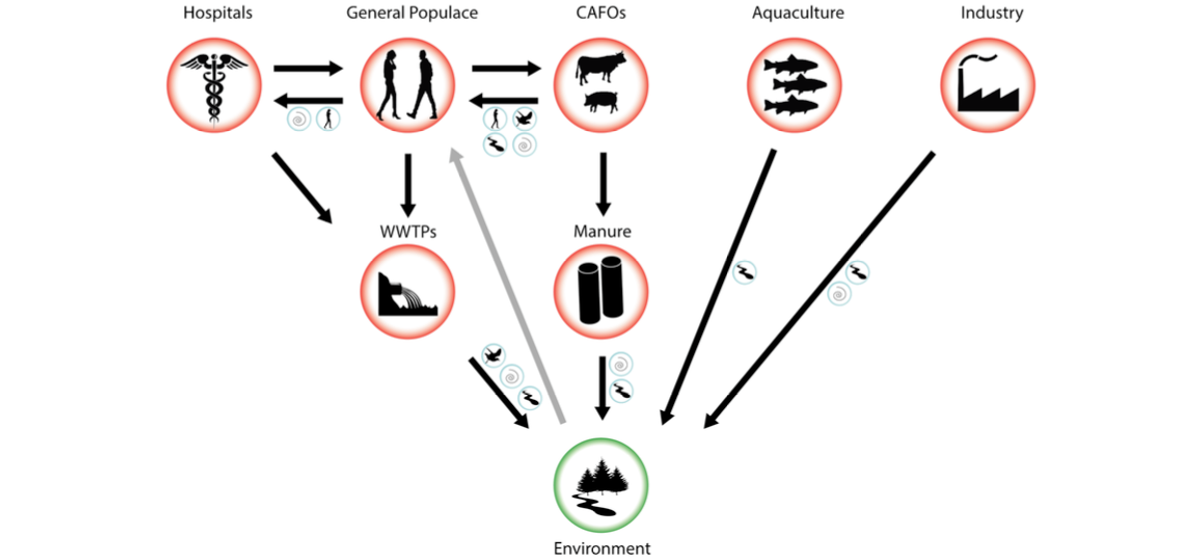
Schematic flow of antibiotic-resistant bacteria (ARB) and antibiotic resistance genes (ARGs) from hotspots of evolution and transmission (red circles) to the environment (green circle). Blue circles indicate possible vectors that may aid transmission between specific environments, including air, surface waters, humans, and other animal vectors. Black arrows indicate known flows of ARB and ARGs. The grey arrow indicates a possible transmission route from a contaminated environment back to the general populace. CAFO: concentrated animal feeding operation; WWTP: wastewater treatment plant (reproduced from Kraemer et al [22], which is published under Creative Commons Attribution 4.0 International License [24]).

Antibiotics can enter the environment through several different routes. Antibiotics and their metabolites are released from hospitals with urine and feces from patients as hospital wastewater effluent [20]. Similarly, antibiotics are released into the wastewater treatment system from people taking antibiotics in their homes [21]. Antibiotics are also used therapeutically or as growth promoters in livestock and poultry in Malaysia. Antibiotics and their metabolites will spread through animal excrement and end up in fields and groundwater, or in the case of antibiotic use in fish farms directly into the aquatic environment [22] (Figure 1). Regardless of the routes for antibiotics disseminating in the environment, it is also likely that resistant bacteria follow the same routes of dispersal [23]. This results in an environment where antibiotics, ARGs, resistant bacteria, and the environmental bacterial flora are mixed. However, the environment is likely becoming a resistance hotspot where ARGs can proliferate and undergo a process of evolution to result in the development of new resistant strains. Pathogens can acquire an ARG from the environmental gene pool by either conjugation, transformation, or transduction. The most important genetic elements capable of being transferred by conjugation are plasmids and integrative conjugative elements, which may carry ARGs and potential virulence genes.

### Scale of the Problem

A total of 88 pathogen-drug combinations were used to quantify the worldwide burden of drug-resistant diseases in 2019. This resulted in an estimated 495 million fatalities, of which 127 million deaths were directly attributed to drug resistance [25]. By 2050, it is estimated that approximately 10 million lives will be at risk annually as a consequence of the rise in drug-resistant infections [26].

Furthermore, antibiotic resistance is a substantial economic burden worldwide. In 2006, approximately 50,000 Americans died due to two common hospital-acquired infections (HAIs), namely pneumonia and sepsis, costing approximately US $8 billion [27]. In Europe, the levels of antibiotic resistance are varied. The common causes of infections are used as indicators to focus on AMR in *S. aureus*, specifically against methicillin (ie, MRSA) as a common cause of severe infections in health facilities and the community. MRSA is among the resistant pathogens that are widely recognized to pose an urgent or serious threat to human health. CRE such as *K*. *pneumoniae and E. coli* are also recognized as major causes of HAIs, including pneumonia. The WHO has listed MRSA as a high-priority (priority 2) pathogen for research and development of new antibiotics, while CRE have been listed as critically important (priority 1) [28].

In Asia, India has been documented as one of the largest burdens of drug-resistant pathogens worldwide, including the highest burden of multidrug-resistant tuberculosis, along with alarmingly high resistance rates among gram-negative and gram-positive bacteria even to newer antimicrobials such as carbapenems and faropenem since its introduction in 2010 [29]. Regional studies in India reported high AMR among pathogens such as *Salmonella typhi*, *Shigella*, *Pseudomonas,* and *Acinetobacter* [30].

In Thailand, approximately 88,000 infections were attributed to AMR, resulting in at least 3.24 million additional days in the hospital and 38,000 deaths [31]. The Global Antimicrobial Resistance Surveillance System (GLASS) launched by the WHO was implemented in Siriraj Hospital in Bangkok, reporting that in-hospital mortality was significantly higher in patients with antibiotic-resistant bacteremia than in patients with antibiotic nonresistant bacteremia (40.5% vs 28.5%, *P*<.001) [32]. The same study concluded that patients with antibiotic-resistant bacteremia utilized more resources than those with antibiotic nonresistant bacteremia [32].

MRSA emerged in Singapore in the 1970s [33], rapidly becoming endemic in local health care facilities, with 35.3% of all *S. aureus* isolates reported to be methicillin-resistant in 2006 [34]. In another study, the prevalence of MRSA was found to be significantly higher in intermediate (29.9%) and long-term (20.4%) care facilities than in acute-care hospitals (11.8%) [35], and the organism has also been sporadically reported in the community as well as in local retail food and animals [36-38].

Nonetheless, there are limited studies examining the role of hospital effluents in contributing to the development of drug-resistant pathogens in the environment. Hospitals are the major users of antibiotics and may become one of the major contributors to AMR in the environment. Furthermore, the levels of antibiotic resistance in hospital effluents may be different from those of other water environments such as homes or agriculture. Therefore, the general aim of this study is to determine the prevalence of antibiotic-resistant pathogenic bacteria and the level of antibiotic residues in hospital effluents in Selangor, Malaysia. This study will also be determining the prevalence of ARB (specifically ESKAPE) present in the effluents of hospitals with an individual sewage treatment system, the occurrence of ARGs among ESKAPE bacteria, and the residual concentrations of antibiotics based on their usage at the selected hospitals. The findings from this study will provide important scientific evidence relevant to stakeholders that can help to reduce or interrupt the spread of AMR from clinical settings into the environment.

## Methods

### Study Design

**Figure 2:**
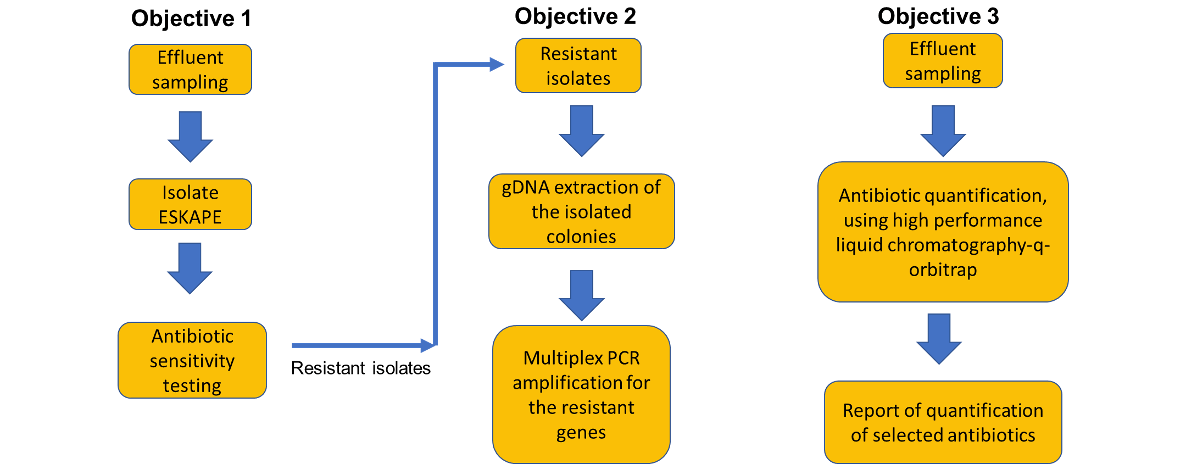
Summary of the workflow for this study. ESKAPE: Enterococcus faecium, Staphylococcus aureus, Klebsiella pneumoniae, Acinetobacter baumannii, Pseudomonas aeruginosa, Enterobacteriaceae; gDNA: genomic DNA; PCR: polymerase chain reaction.

A cross-sectional study will be performed at preidentified hospitals with three specific objectives as depicted in the workflow diagram in Figure 2. Objective 1 is to obtain resistant isolates of ESKAPE bacteria from the hospital effluent and to perform antibiotic susceptibility testing (AST). Objective 2 is to extract the genomic DNA of the resistant isolates from objective 1 to identify the isolates and the ARGs present in the isolates. Objective 3 is to quantify the level of targeted antibiotic residues from the hospital effluent samples. The targeted antibiotics that will be quantified are ceftriaxone, ciprofloxacin, meropenem, vancomycin, colistin, piperacillin, and tazobactam, which are the most highly used antibiotics in the hospital setting. The outcome of activities in objective 1 and 2 will provide estimates of the prevalence of the ARB in the hospital effluent, while the outcome of objective 3 will determine the level of antibiotic residues in the hospital effluent to address the overall aim of this study.

### Selection of Study Area

We will identify all the tertiary hospitals in Selangor, one of the states in Malaysia, prior to effluent sample collection, based on the following inclusion and exclusion criteria.

**Figure 3:**
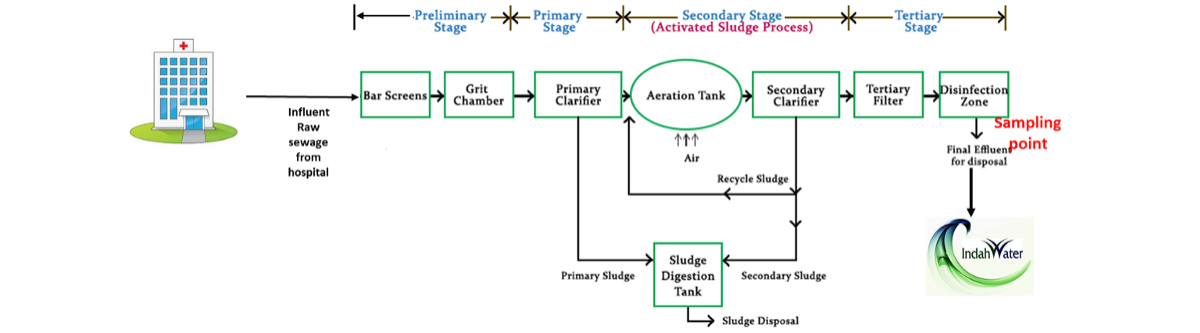
The sampling point for the effluent sample to be collected is at the final effluent for the disposal outlet, as depicted in the diagram. This is the outlet after the treatment before entering the river system that is going to be treated by the Indah Water Treatment Plant to be used as a clean water source for the public.

**Figure 4:**
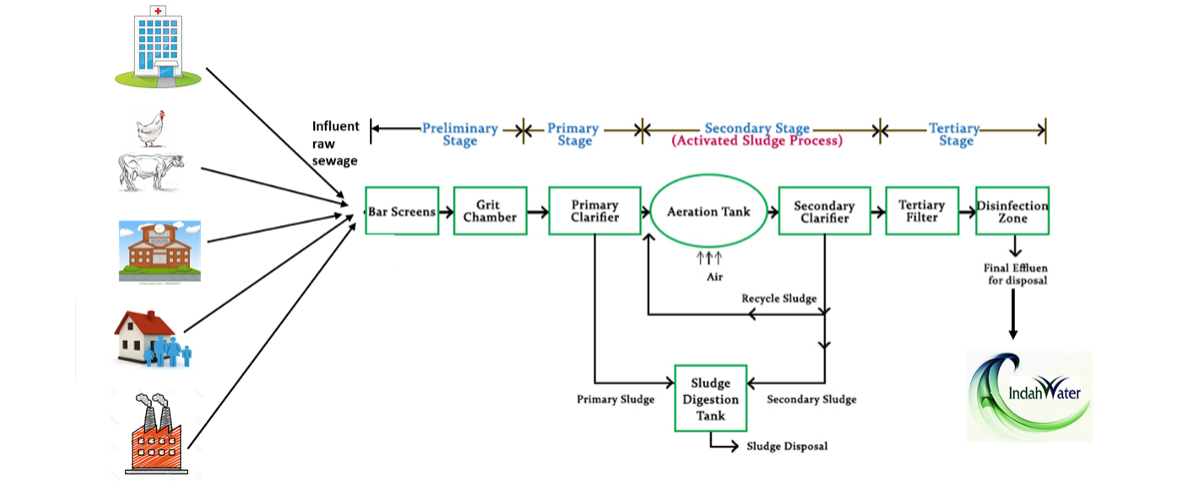
The waste from multiple human activities such as farming, domestic, school, factory, and hospital activities is concentrated into same sewage treatment plant before release to the river to be treated by Indah Water Treatment Plant as a clean water source for the public.

Hospitals with individual sewage treatment plants will be included. An individual sewage treatment plant is a system whereby the sewage from the hospital is the only source that enters the sewage treatment plant, as depicted in Figure 3. Hospitals with nonindividual sewage treatment plants will be excluded. A nonindividual sewage treatment plant is defined as a communal sewage treatment plant whereby the sewage entering the sewage treatment plant is coming from various sources such as housing, farming, and manufacturing industries, as illustrated in Figure 4.

### Sample Size Calculation

Calculation of sample size is based on the prevalence using the following formula [39]: *N*=*Z^2^p*(1 – *p*)/*d^2^*, where *N* is the sample size, *Z* is the statistic for a level of confidence, and *p* is the expected prevalence or proportion (considering a total proportion of 100%, if 20% then *p*=0.2), and *d* is precision. For this study in proportion of 100%, if 5%, *d*=0.05. Considering a conventional 95% CI as the level of confidence, the *Z* value is 1.96. The sample size was determined from prevalence estimates of previous studies of ESKAPE isolates from the hospital or wastewater treatment plant with the 95% CIs, as shown in Table 1. This calculation served as the guidance for the number of plates to be used to isolate the targeted ESKAPE bacteria.

**Table 1:** Prevalence of antibiotic-resistant bacteria from related studies.

Study and bacterial species			Prevalence, %		Sample size (number of plates to isolate ESKAPE^a^ bacteria)
**Haller et al [40]**					
	*Pseudomonas* spp.	28.2		312	
	*Klebsiella* spp.	28.2		312	
	*Enterobacter* spp.	18.3		230	
	*Citrobacter* spp.	11.3		155	
Hrenovic et al [41]: *Acinetobacter baumannii*			57.0		377
Thompson et al [42]: methicillin-resistant *Staphylococcus aureus*			81.0		237
**Varela et al [43]**					
	*Enterococcus* spp. on vancomycin	0.7		11	
	*Enterococcus* spp. on ciprofloxacin	3.4		54	
**Karimi et al [44]**					
	*Enterococcus faecium*	6.03		88	
	*Enterococcus faecalis*	0.63		10	

^a^ESKAPE: *Enterococcus faecium*, *Staphylococcus aureus*, *Klebsiella pneumoniae*, *Acinetobacter baumannii*, *Pseudomonas aeruginosa*, and *Enterobacter.*

### Sample Collection and Preparation

In total, two bottles of effluent water will be collected. Samples for objective 1 and objective 2 will be collected in a 1-L sterile screw-capped Schott Duran (Germany) amber bottle. In addition, samples for antibiotics quantification (objective 3) will be collected in a 1-L high-density polyethylene (Kris Plastic Industries, Malaysia) sample bottle. All samples will be kept in an ice-packed cooler box maintained at 4°C and transported back to the Environmental Microbiology Laboratory, Institute for Medical Research, for immediate processing. The samples will be collected at the final effluent disposal point of the sewage treatment system before reaching the Indah Water Treatment Plant Malaysia (Figure 3).

### Microbiological Analysis

#### Isolation of ESKAPE ARB

**Figure 5:**
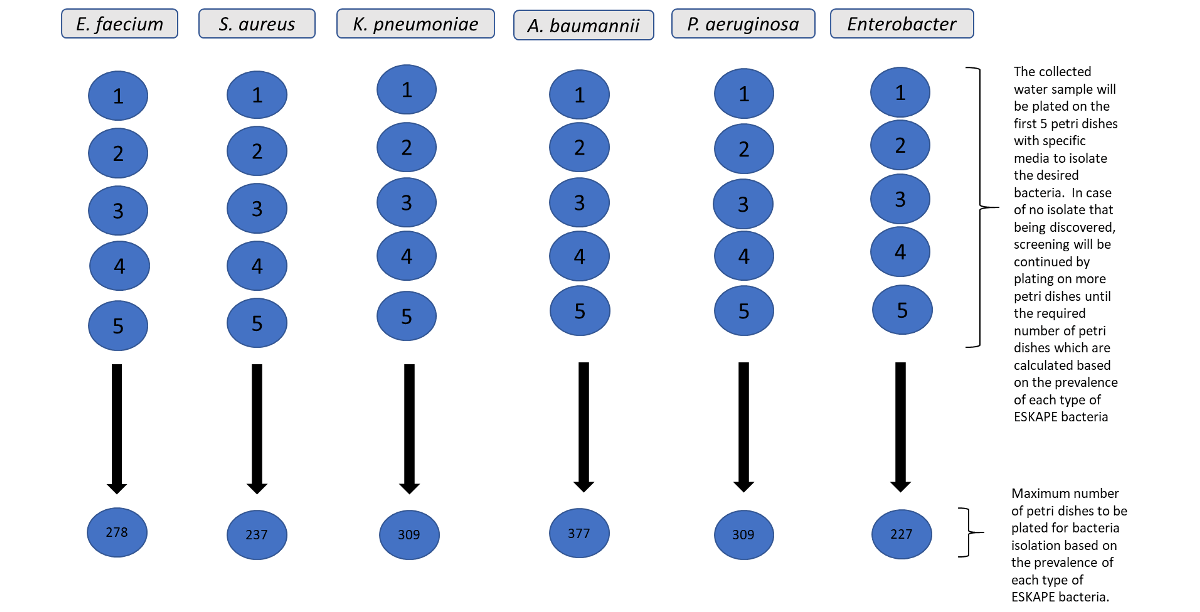
The number of plates to be used to isolate ESKAPE (Enterococcus faecium, Staphylococcus aureus, Klebsiella pneumoniae, Acitenobacter baumannii, Pseudomonas aeruginosa, and Enterobacter) pathogens from the hospital effluent based on the calculated sample size. See Table 2 for the selective media used for each species.

Ten-fold serial dilutions for each water sample will be prepared in 50-mL sterile saline solution and 10 mL of the sample will be filtered through sterile 0.45-µm pore size cellulose acetate filters (Sartorious Singapore Pte Ltd) in triplicate (Figure 5). The filter will be then placed onto the selective media to isolate ESKAPE pathogens. The selective media to be used in this process of isolation are listed in Table 2. Identification of the ESKAPE bacteria will be carried out using the 16S RNA polymerase chain reaction (PCR) technique with a universal primer pair (forward, CCT ACG GGA GGC AGC AG; reverse, ATT ACC GCG GCT GCT GG) under the following condition: polymerase activation at 94°C for 2 minutes, denaturation at 94°C for 5 seconds, annealing at 60°C for 10 seconds, extension at 72°C for 20 seconds, and holding at 4°C.

The positive colonies will be subjected to AST using the disk diffusion method (Kirby Bauer) referring to the Clinical Laboratory Standards Institute (CLSI) guidelines. The antibiotics that will be used for AST are ceftriaxone (30 µg), ciprofloxacin (5 µg), meropenem (10 µg), vancomycin (30 µg), colistin (10 µg), and piperacillin/tazobactam (110 µg). For the bacteria that are not available in CLSI guidelines, measurement will refer to European Committee of Antimicrobial Susceptibility Testing (EUCAST) guidelines. The minimal inhibitory concentration of colistin will be determined using a liquid broth dilution test as recommended by EUCAST with cation-adjusted Mueller-Hinton broth (NutriSelect Plus, Germany), because the disk diffusion method is not reliable for this antibiotic since its large molecular size results in poor diffusion into the agar medium, leading to inaccurate results with a high error rate and poor reproducibility [45].

**Table 2:** Selective media and the incubation condition to isolate pathogens from the effluent samples.

Selective organism	Medium	Incubation condition
*Enterococcus faecium*	Chromogenic *E. faecium* Agar (Himedia, India)	37°C, aerobic, 24-48 hours
*Staphylococcus aureus*	Mannitol Salt Agar (BD, Germany)	37°C, aerobic, 24-48 hours
*Klebsiella pneumoniae*	Eosin Methylene Blue Agar Modified (HHT) (BD-BBL, USA)	37°C, aerobic, 24-48 hours
*Acinetobacter baumannii*	Chromagar Acinetobacter (CHROMagar, Paris France)	37 °C, aerobic, 24-48 hours
*Pseudomonas aeruginosa*	Cetrimide Agar (Neogen USA)	37°C, aerobic, 24-48 hours
*Enterobacter*	MacConkey II Agar (BD-BBL, USA)	37°C, aerobic, 24 hours

#### Detection of ARGs Among ESKAPE Isolates

Each colony found to be resistant to any of the tested antibiotics will be cultured and inoculated in tryptone soy broth medium (CM0129, Oxoid). The culture will be incubated at 37°C for 18-24 hours. The genomic DNA will be extracted using the PureGene Bacteria Kit (Qiagen, Germany) following the manufacturer’s instructions. The concentration of the extracted DNA will be measured using a Multiskan SkyHigh microplate spectrophotometer (Thermo Scientific) before multiplex PCR amplification. Primers were selected to amplify the targeted ARGs based on published findings [46-56] (see Multimedia Appendix 1). The extracted DNA will be amplified using multiplex PCR following the single-template PCR method. The PCR conditions will be optimized according to published methods. The size of the amplified products will be confirmed by 3% agarose gel (NextGene) electrophoresis at 90 V for 90 minutes. The PCR products will be sent to an outsourcing service for Sanger sequencing. The sequencing results will be analyzed using the Basic Local Alignment Search Tool nucleotide (BLASTn) tool in the National Center for Biotechnology Information database to identify the correct amplified relevant genes. 

### Chemical Analysis

#### Quantification of the Concentrations of Selected Antibiotics

An ultrahigh-performance liquid chromatography (UHPLC) system will be used to quantify the levels of antibiotics from all samples following the method reported by Kim et al [57]. Samples will be collected and treated by preconcentration using a nitrogen evaporator. The samples will then be cleaned by an automated solid-phase extraction system. The analysis will be performed using Q-exactive liquid chromatography-mass spectrometry. External standards of antibiotics will include ceftriaxone, ciprofloxacin, meropenem, vancomycin, colistin, piperacillin, and tazobactam. The internal standards will be ciprofloxacin-d8 (molecular weight: 339.38) and piperacillin-d5 (molecular weight: 522.59). The analytical column will be Waters Xbridge C18 Column (50 mm × 2.1 mm, 2.5 µm inner diameter) run at 35°C. Two types of solvents will be used: 0.1% formic acid (solvent A) in water and 100% acetonitrile (solvent B). The solvent gradients with a flow rate of 0.2 mL/min for this experiment are shown in Table 3.

**Table 3:** Solvent gradients with a flow rate of 0.2 mL/min.

Time (minutes)	Solvent A (0.1% formic acid), %	Solvent B (100% acetonitrile), %
0.0	98	2
4.9	35	65
13.5	2	98
21.0	2	98
21.5	98	2
26.0	98	2

### Data Analysis

The prevalence of ARB will be calculated using the following formula:

Prevalence (%)=(number of resistant isolates/number of resistant isolates + number of susceptible isolates) × 100

To detect ARGs carried by ESKAPE bacteria, nucleotide results from the sequencing will be checked using Chromas, a DNA sequencing software to view and edit the chromatograms (traces) from automated Sanger sequencers. The edited chromatograms from the sequencing results will be sent to the BLASTn database to confirm the amplified genes.

The concentration of the antibiotic residues quantified using UHPLC will be compared to the antibiotic usage in the hospitals.

### Ethics Considerations

No primary data will be collected and thus no ethical approval is required. The study protocol was approved by the Medical Research and Ethical Committee, Ministry of Health Malaysia. This study is also registered under the National Medical Research Registry (NMRR-19-3637-51680) Malaysia.

## Results

The primary outcome measure for this study is the prevalence of resistant ESKAPE bacteria isolated from antibiotic-selective media. The bacteria will be identified by 16S RNA PCR to determine species. The secondary outcome of this study is the occurrence of ARGs that may be discovered from the resistant ESKAPE bacteria isolates as the likely cause of the resistance toward the antibiotics (Table 2). The multiplex PCR method will be applied to screen the ARGs present. As of July 2022, effluent sampling has been conducted in three hospitals and analysis of samples is ongoing to determine the level of antibiotic residues. ESKAPE bacteria were isolated and AST is ongoing to determine the resistant isolates. Some preliminary findings for *E. faecium* from Hospital A showed that 80% (8/10) were resistant to vancomycin and 20% (2/10) were susceptible, whereas only 10% (1/10) were resistant, 60% (6/10) had intermediate resistance, and 30% (3/10) were susceptible to ciprofloxacin. Moreover, 100% (10/10) of *E. faecium* isolates from Hospital B were resistant toward vancomycin but susceptible toward ciprofloxacin. Resistant isolates will be processed for further screening of ARGs using PCR. Effluent sampling activities were suspended for a few months due to the COVID-19 pandemic, which caused a delay in the downstream analysis. The findings of the study will be disseminated through peer-reviewed journals and scientific conferences.

## Discussion

VRE represent an issue of both medical and public health importance, as these bacteria are commonly associated with multidrug-resistant infections and persistent colonization in patients [58]. From our early findings, the prevalence of vancomycin-resistant *E. faecium* from two hospitals was more than 80%, which was higher than the prevalence of 11% reported in a study performed in the Czech Republic on isolates collected from Military Hospital Olomouc and University Hospital Olomouc [59].

The outcomes of this study will fill the gap of important information for clinical practitioners to determine whether the hospitals in Malaysia are main contributors to the AMR problem in the country. This study will provide baseline information on the prevalence of antibiotic resistance from hospital effluents in Malaysia to assist in hospital wastewater management and improve antibiotic stewardship in Malaysia.

This study will have an impact on the improvement of hospital sewage treatment because the data will provide information to the Engineering Service Division of the Ministry of Health that reflects the efficiency of the current sewage treatment in Ministry of Health hospitals. This should facilitate the hospital’s sewage manager in the Engineering Service Division in effective service improvement.

Moreover, the findings from this study will help in the optimization of antimicrobial stewardship programs by recommending strategies for infectious disease physicians and pharmacists in their prospective audit and feedback of antimicrobial prescriptions to clinicians, formulary restriction, education, and guidelines. Antimicrobial stewardship programs are aiming to optimize antimicrobial selection, dosing, route, and duration of therapy to maximize a clinical cure or prevention of infection, while limiting unintended consequences such as the emergence of resistance, adverse drug events, and extra costs.

Ultimately, this study will contribute to the improvement in public health. The improvement in the hospital sewage system will eventually lead to controlling antibiotics pollution in the environment by reducing the spread of clinically driven ARB and ARGs into the environment. This will in turn reduce the unnecessary demand for stronger antibiotics or even failure to treat many types of resistant infections.

## Appendix

Multimedia Appendix 1 Published primers representing the occurrence of bacterial DNA and antibiotic resistance genes.

## Abbreviations

AMR: antimicrobial resistance
ARB: antimicrobial-resistant bacteria
ARG: antibacterial resistance gene
AST: antibiotic susceptibility testing
BLASTn: Basic Local Alignment Search Tool nucleotide
CLSI: Clinical Laboratory Standards Institute
CRE: carbapenem-resistant Enterobacteriaceae
ESKAPE: Enterococcus faecium, Staphylococcus aureus, Klebsiella pneumoniae Acinetobacter baumannii, Pseudomonas aeruginosa, Enterobacteriaceae
EUCAST: European Committee of Antimicrobial Susceptibility Testing
GLASS: Global Antimicrobial Resistance Surveillance System
HAI: hospital-acquired infection
MRSA: methicillin-resistant Staphylococcus aureus
MyAP-AMR: Malaysian Action Plan on Antimicrobial Resistance
PCR: polymerase chain reaction
UHPLC: ultrahigh-performance liquid chromatography
VRE: vancomycin-resistant enterococci
WHO: World Health Organization

## Data Availability

The authors confirm that the data supporting the findings of this study are available within the article and supplementary materials.
