# Supplementary material for: Prevalence of Antibiotic-Resistant Pathogenic Bacteria and Level of Antibiotic Residues in Hospital Effluents in Selangor, Malaysia: Protocol for a Cross-sectional Study
Source: JMIR Res Protoc. 2023 May 29;12:e39022. doi: 10.2196/39022 (PMC10263467; doi:10.2196/39022)
Supplement: Multimedia Appendix 1 [file resprot_v12i1e39022_app1.docx]

Published primers representing the occurrence of bacterial DNA and antibiotic resistance genes.

| Target/antibiotics | | Resistance mechanism | Primer sequence (5′ to 3′) | Size (bp^a^) | PCR^b^ conditions | Reference |
| --- | --- | --- | --- | --- | --- | --- |
| Bacterial 16S rDNA | | Not applicable | Forward: CCTACGGGAGGCAGCAG; reverse: ATTACCGCGGCTGCTGG | 202 | Initial denaturation of 94°C for 5 min, 30 cycles of 94°C for 30 s for denaturing the DNA strands, 53°C for 1 min to anneal the primers to DNA, 72°C for 30 s for extension, and 72°C for 7 min for final extension | Bergeron et al [45] |
| Macrolides, *ermB* | | Ribosomal protection | Forward: GATACCGTTTACGAAATTGG; reverse: GAATCGAGACTTGAGTGTGC | 364 | Initial denaturation of 94°C for 5 min, 30 cycles of 94°C for 30 s for denaturing the DNA strands, 53°C for 1 min to anneal the primers to DNA, 72°C for 30 s for extension, and 72°C for 7 min for final extension | Bergeron et al [45] |
| Penicillin-like (eg, amoxycilin): *mecA* | | Beta-lactam binding protein | Forward: GTAGAAATGACTGAACGTCCG ATAA; reverse: CCAATTCCACATTGTTTCGGT CTAA | 310 | Initial denaturation of 94°C for 10 min, 25 cycles of 94°C for 45 s for denaturing the DNA strands, 55°C for 45 s to anneal the primers to DNA, 72.2 °C for 1 min and 15 s | Bergeron et al [45] |
| **Beta lactams** | | | | | | |
|  | *BlaNDM-1* | Hydrolysis | Forward: GGG CAG TCG CTT CCA ACG GT; reverse: GTA GTG CTC AGT GTC GGC AT | 475 | Initial DNA release and denaturation at 94°C for 5 min; followed by 36 cycles of 94°C for 30 s, 52°C for 40 s, and 72°C for 50 s; and a single, final, elongation step at 72°C for 5 min | Shanthi et al [46] |
|  | *BlaCTX-M* | Hydrolysis | Forward: CTATGGCACCACCAACGATA; reverse: ACGGCTTTCTGCCTTAGGTT | 103 | Initial DNA denaturation 95ºC 3 min (1 cycle); denaturation of DNA 95°C for 15 s and annealing at 60°C for 20 s (40 cycles) | Marti et al [47] |
|  | *BlaOXA-48* | Hydrolysis | Forward: TTGGTGGCATCGATTATCGG; reverse: GAGCACTTCTTTTGTGATGGC | 585 | Initial denaturation at 95°C for 5 min, 35 cycles at 95°C for 1 min, annealing at 56°C for 1 min and 72°C for 1 min, followed by a single, final elongation step at 72°C for 5 min | Mlynarcik et al [48] |
|  | *BlaSHV* | Hydrolysis | Forward: CGCTTTCCCATGATGAGCACC TTT; reverse: TCCTGCTGGCGATAGTGGAT C TTT | 110 | Initial DNA denaturation at 95°C for 3 min (1 cycle); denaturation of DNA at 95°C for 15 s and annealing at 64°C for 30 s (40 cycles) | Marti et al [47] |
|  | *VanA* | Amino acid cleavage | Forward: GGCAAGTCAGGTGAAGAT G; reverse: ATCAAGCGGTCAATCAGT TC | 713 | 5 min at 94°C for initial enzyme activation, followed by 40 cycles of amplification consisting of denaturation at 94°C for 1 min, annealing at 55°C for 1 min, and extension at 72°C for 2 min, with a final extension at 72°C for 5 min | Azimian et al [49] |
|  | *VanA* | Amino acid cleavage | Forward: AATACTGTTTGGGGGTTGC TC; reverse: TTTTTCCGGCTCGACTTCC T | 734 | Initial denaturation of 10 min at 94°C; followed by 35 cycles of denaturation at 94°C for 1 min, annealing at 58°C for 1 min, and extension at 72°C for 1 min; and final extension at 72°C for 10 min | Kafil and Asgharzadeh [50] |
|  | *VanB* | Amino acid cleavage | Forward: GTGACAAACCGGAGGCGAG GA; reverse: CCGCCATCCTCCTGCAAAAA A | 430 | Initial denaturation step at 94°C for 10 min; followed by 30 cycles of 94°C for 30 s, 50°C for 45 s, and 72°C for 30 s; and a final elongation step at 72°C for 10 min | Saadat et al [51] |
|  | *VanC1* | Modified peptidoglycan | Forward: GGTATCAAGGAAACCTC; reverse: CTTCCGCCATCATAGCT | 822 | 94°C for 2 min for the first cycle; 94°C for 1 min, 54°C for 1 min, and 72°C for 1 min for the next 30 cycles; and 72°C for 10 min for the last cycle | Praharaj et al [52] |
| **Colistin** | | | | | | |
|  | *mcr-1* | Horizontal gene transfer | Forward: GCTCGGTCAGTCCGTTTGTTCT TG; reverse: GGATGAATGCGGTGCGGTCTT | 1497 | 3 min at 93°C; followed by 35 cycles consisting of 15 s at 93°C, 30 s at 57°C, 70 s at 68°C; and one extension cycle at 72°C for 5 min | Zhang et al [53] |
|  | *mcr-2* | Horizontal gene transfer | Forward: AGCCGAGTCTAAGGACTTG ATGAATTT; reverse: GCGGTATCGACATCATAGT CATCTTG | 576 | 3 min at 93°C; followed by 35 cycles consisting of 15 s at 93°C, 30 s at 57°C, 70 s at 68°C; and one extension cycle at 72°C for 5 min | Zhang et al [53] |
|  | *mcr-3* | Horizontal gene transfer | Forward: CGCTTATGTTCTTTTTGGCA CTGTATT; reverse: TGAGCAATTTCACTATCGA GGTCTTG | 1063 | 3 min at 93°C; followed by 35 cycles consisting of 15 s at 93°C, 30 s at 57°C, 70 s at 68°C; and one extension cycle at 72°C for 5 min | Zhang et al [53] |
| **Integrons** | | | | | | |
|  | Intl1 | Capable of capturing gene cassettes and play key roles in the dissemination and spread of resistance genes | Forward: CCTCCCGCACGATGATC; reverse: TCCACGCATCGTCAGGC | 280 | Annealing temperature: 55 °C | Goldstein et al [54] |
|  | Intl2 | Capable of capturing gene cassettes and play key roles in the dissemination and spread of resistance genes | Forward: TTATTGCTGGGATTAGGC; reverse: ACGGCTACCCTCTGTTATC | 233 | Annealing temperature: 50 °C | Goldstein et al [54] |
|  | *qnrA* | Plasmid-mediated quinolone resistance | Forward: TCAGCAAGAGGATTTCTCA; reverse: GGCAGCA CTATTACTCCCA | 627 | 94°C for 45 s, 48°C for 45 s, and 72°C for 45 s for 30 cycles. | Wang et al [55] |

^a^bp: base pair.

^b^PCR: polymerase chain reaction.
